# Variations in IL-23 and IL-25 receptor gene structure, sequence and expression associated with the two disease forms of sheep paratuberculosis

**DOI:** 10.1186/s13567-016-0314-4

**Published:** 2016-02-09

**Authors:** Louise Nicol, Anton Gossner, Craig Watkins, Francesca Chianini, Robert Dalziel, John Hopkins

**Affiliations:** The Roslin Institute and R(D)SVS, University of Edinburgh, Easter Bush, Midlothian, EH25 9RG UK; Moredun Research Institute, International Research Centre, Pentlands Science Park, Penicuik, Midlothian, EH26 0PZ UK

## Abstract

The immunopathology of paucibacillary and multibacillary sheep paratuberculosis is characterized by inflammatory T cell and macrophage responses respectively. IL-23 and IL-25 are key to the development of these responses by interaction with their complex receptors, IL-23R/IL-12RB1 and IL-17RA/IL-17RB. In humans, variations in structure, sequence and/or expression of these genes have been implicated in the different pathological forms of tuberculosis and leprosy, and in gastrointestinal inflammatory disorders such as Crohn’s disease. Sequencing has identified multiple transcript variants of sheep *IL23R*, *IL12RB1* and *IL17RB* and a single *IL17RA* transcript. RT-qPCR assays were developed for all the identified variants and used to compare expression in the ileo-caecal lymph node of sheep with paucibacillary or multibacillary paratuberculosis and uninfected animals. With IL-23 receptor, only the *IL12RB1v3* variant, which lacks the receptor activation motif was differentially expressed and was significantly increased in multibacillary disease; this may contribute to high Th2 responses. Of the *IL17RB* variants only full length *IL17RB* was differentially expressed and was significantly increased in multibacillary pathology; which may also contribute to Th2 polarization. *IL17RA* expression was significantly increased in paucibacillary disease. The contrast between the *IL17RA* and *IL17RB* results may indicate that, in addition to Th1 cells, Th17 T cells are also involved in paucibacillary pathology.

**Electronic supplementary material:**

The online version of this article (doi:10.1186/s13567-016-0314-4) contains supplementary material, which is available to authorized users.

## Introduction

Paratuberculosis (Johne’s disease) is an endemic enteric disease of ruminants caused by *Mycobacterium avium* subspecies *paratuberculosis* (MAP) [1]; and like human mycobacterial infections, clinical paratuberculosis develops in a minority of infected animals [2]. In sheep, approximately one-third of the diseased animals develop paucibacillary or tuberculoid pathology and two-thirds develop multibacillary or lepromatous disease [2]; with lesions localized largely in the lamina propria of the terminal ileum [2]. Paucibacillary pathology is characterized by lymphocyte infiltration, granulomatous inflammation and few bacteria. In contrast multibacillary lesions are composed largely of heavily infected macrophages [2]. There is progression from sub-clinically infected to paucibacillary disease and terminal multibacillary pathology [3]; however progression occurs less frequently in sheep than in cattle and paucibacillary disease is usually fatal in infected sheep [2, 4, 5].

The immunology of paratuberculosis in sheep is also similar to tuberculoid and lepromatous forms of the human mycobacterial diseases, tuberculosis and leprosy [6, 7]. Paucibacillary disease is associated with a highly polarized Th1/Th17 response, characterized by high levels of IL-12, IL-17A and IFNγ [8–10]; and multibacillary paratuberculosis is linked with a strong Th2 response with high levels of IL-5 and IL-10 [8–11]. MAP has also been implicated as an aetiological agent of inflammatory intestinal diseases in humans because of the apparent similarity between the pathology of paucibacillary paratuberculosis and Crohn’s disease [12]. Like the human mycobacterial diseases, the epidemiology of paratuberculosis strongly suggests a host genetic susceptibility to disease severity and pathological form [13, 14]. Many of the genes associated with severity of human disease and pathology belong to the pathways that control differential T cell activation [13]; and genes in these pathways are also linked to gastrointestinal inflammatory diseases including ulcerative colitis and Crohn’s disease [12–15].

Of particular interest in this study are genes for the cytokines and cytokine receptors that control the expression of Th1, Th17 and Th2 subsets associated with the different forms of chronic gastrointestinal inflammation [15, 16] and are also important in the pathogenesis of tuberculosis and leprosy [7–13]. The activation of Th1/Th17 T cells is regulated by the interaction of the related heterodimeric cytokines IL-12 and IL-23 with their respective receptor complexes, IL-12RB1/IL-12RB2 and IL-12RB1/IL-23R [17]. Macrophages play a critical role in the presentation of antigen to T cells and their downstream activation and polarization, and are the target cells for MAP infection [18]. Mycobacterial infection of macrophages affects the expression of IL-12 and IL-23 [8, 19, 20] and therefore potentially influences the polarization of the immune response and consequently disease pathology. In contrast, the activation of Th2 T cells is influenced by IL-25 [21], which is produced by cells such as intestinal epithelium and innate lymphoid cells 2 (ILC2) and functions by interacting with its complex receptor IL-17RA/IL-17RB on T cells [22].

Variations in gene structure, sequence or expression levels of these cytokines and their receptors have been implicated in human mycobacterial pathogenesis and/or gastrointestinal and respiratory inflammatory diseases [13, 23]. Genome wide association studies have identified the association of single nucleotide polymorphisms (SNPs) of *IL12B* and *IL23R* with susceptibility to leprosy and tuberculosis [13, 24] and with Crohn’s disease [16]. In addition, specific splice variants of *IL12B* and *IL23R* have been linked to disseminated BCG infection [25] and with inflammatory bowel disease [26]. Studies on IL-25 and its receptor complex have found similar associations with inflammation; SNP analysis of *IL17RA* has linked three alleles associated with aspirin exacerbated respiratory disease [27] and *IL17RB* polymorphisms with asthma [28].

The aims of this study were to characterize the different sequence and transcript variants of the sheep IL-23 and IL-25 receptor complexes associated with Th1/Th17 and Th2 T cell polarization within the ileo-caecal lymph node (ICLN), the major immune inductive site for pathogens infecting the terminal ileum [29]; and to assess the expression of the different transcript variants in relation to defined paucibacillary and multibacillary pathology of sheep paratuberculosis.

## Materials and methods

### Animals, disease diagnosis and tissue collection

Animals with clinical paratuberculosis were out-bred Blackface or Blackface cross female sheep with naturally acquired MAP infection sourced from six farms; uninfected controls were from a single source with no history of paratuberculosis. The pathology of paratuberculosis was confirmed in clinically-affected sheep at post-mortem, by haematoxylin and eosin and Ziehl–Neelsen histopathology of the terminal ileum, MLN and ICLN. Infection was confirmed in affected sheep by *IS900* PCR; all control sheep were *IS900* negative (Table 1). There were six animals in each of three groups. Tissue was ICLN, removed immediately post-mortem, cut into blocks of ~0.5 g and placed in five volumes of RNAlater (Ambion, Huntingdon, UK), which were then incubated overnight at 4 °C and stored at −80 °C. No animals were euthanized specifically for this study; the animals were humanely culled for clinical reasons of paratuberculosis disease or for reasons unrelated to this study (uninfected controls).

**Table 1:** **Breed, age, histopathology and disease diagnosis of sheep**

Sheep ID	Breed	Origin^a^	Age (years)	SGI^b^	AFB^c^	Tissue specific lesion grade^d^	*IS900* ^e^	Diagnosis^f^
SH.139	Blackface	A	3	5	4	Severe	+, +	Multibacillary
SH.140	Blackface	A	3	7	4	Severe	+, +	Multibacillary
SH.146	Blackface x	B	2.5	5	4	Severe	+, +	Multibacillary
SH.190	Blackface	C	3	5	4	Severe	+, +	Multibacillary
SH.199	Blackface	C	2	6	4	Severe	+, +	Multibacillary
SH.204	Blackface	D	1.5	6	4	Severe	+, +	Multibacillary
SH.107	Blackface x	E	2.5	2.5	0	Mild	+, +	Paucibacillary
SH.147	Blackface x	B	2	3	0	Mild	+, +	Paucibacillary
SH.155	Blackface	F	3	2	0	Mild	+, +	Paucibacillary
SH.160	Blackface x	B	3	2.5	0	Mild	+, +	Paucibacillary
SH.188	Blackface x	B	4	4	1	Moderate	+, +	Paucibacillary
SH.205	Blackface	D	4	2	0	Mild	+, +	Paucibacillary
K207	Blackface	G	2.5	0	0	ND	−, −	Control
K208	Blackface	G	2.5	0	0	ND	−, −	Control
K213	Blackface	G	2.5	0	0	ND	−, −	Control
K224	Blackface	G	2.5	0	0	ND	−, −	Control
K227	Blackface	G	2.5	0	0	ND	−, −	Control
K229	Blackface	G	2.5	0	0	ND	−, −	Control

^a^Source farms.

^b^Severity of granulomatous inflammation (SGI) grading: based on total number of epithelioid macrophages and leukocyte distribution patterns of the terminal ileum [5].

^c^Acid fast bacteria (AFB) -; grading: grades 0–2 were defined as paucibacillary; grades 3–4 were defined as multibacillary observed in terminal ileum tissue [5].

^d^Tissue specific lesion grading: for each terminal ileum histological section, the sum of the points (SGI + AFB) was used to determine tissue-specific lesion grades. A severity of “none” was assigned to those with a lesion grade of 0, “mild” was assigned to a lesion grade of ≥2 and ≤3, “moderate” was assigned to those with a lesion grade of >3 and ≤5 and “severe” was assigned to a lesion grade of 6–11.

^e^ *IS900* PCR result using each of the two primer sets.

^f^Diagnosis: based on histopathological observations.

### *IS900* quantitative PCR

ICLN of all animals was tested for the presence of MAP by PCR for insertion sequence *IS900*. Two independent primer sets were used [30, 31]; set 1 generated an amplicon of 99 bp (for: GTTCGGGGCCGTCGCTTAGG; rev: GCGGGCGGCCAATCTCCTT) and set 2 generated a product of 314 bp (for: CTGGCTACCAAACTCCCGA; rev: GAACTCAGCGCCCAGGAT). Genomic DNA (gDNA) was purified using the Wizard^®^ Genomic DNA Purification Kit (Promega, UK). All reactions used FastStart Taq DNA Polymerase (Roche Diagnostics, UK) following the manufacturer’s instructions, with 500 ng of gDNA and 0.2 μM of each primer. PCRs were performed using a Veriti^®^ Thermal Cycler (Applied Biosystems) with four separate reactions performed for each primer set at different annealing temperatures. PCR reactions consisted of a heat activation step for 5 min at 95 °C, 35 cycles of 15 s at 95 °C, 15 s at 55, 58, 60 and 62 °C, and 30 s at 72 °C, with a final elongation step of 10 min at 72 °C.

PCR products were analysed on a 2% agarose gel electrophoresis, purified with a MinElute Gel Extraction Kit (Qiagen, UK) according to the manufacturer’s instructions; cloned using a TOPO TA cloning kit for sequencing (Invitrogen) and sequenced using BigDye Terminator v3.1 Cycle Sequencing Kit and 3730 DNA Analyser (Applied Biosystems). The presence of an amplicon, confirmed by sequencing, from either primer set was taken as a positive result for the presence of MAP; the absence of an amplicon in all PCR reactions was confirmation of absence of MAP infection. All multibacillary and paucibacillary animals were positive and all uninfected, control animals were negative for *IS900*.

### Cloning of ovine gene fragments

RNA was isolated from ~20 mg of ICLN using the Ribopure Kit (Ambion, UK) according to the manufacturers’ instructions. Genomic DNA was removed by On-column PureLink^®^ DNase I treatment (Ambion). RNA quantity, quality and integrity were determined using a Labtech NanoDrop ND-1000 spectrophotometer and Agilent 2200 TapeStation system; samples had an RNA Integrity Number of 7.4–9. cDNA was synthesised from 1.0 μg RNA using SuperScript™ II RT with RNaseOUT (Invitrogen, UK) and oligo-dT(15) primer (Promega, UK), in 20 μL final volume. The predicted sequences of the sheep genes were obtained by NCBI-BLAST of the bovine (or if bovine sequences were not available, human and/or mouse) sequences against the Oar v3.1 sheep genome assembly [32]. Primers were selected using Primer-BLAST [33] and reanalysed using Net Primer [34]. The primers were used to amplify overlapping sections of genes, with at least 100 bp overlap of amplicons; each primer set was used in RT-PCR using FastStart Taq (Roche, UK) as per manufacturer’s instructions. New primers were selected (Additional file 1) from these assembled sequences to obtain full length transcripts using Platinum^®^*Taq* DNA Polymerase High Fidelity (Invitrogen). PCR products were purified using a MinElute PCR Purification Kit (Qiagen), ligated into pGEM^®^-T Easy Vector (Promega). Plasmids were extracted using QIAprep Spin MiniPrep Kit (Qiagen) following the manufacturers’ protocol. At least three clones from all diseased sheep, for each insert, were sequenced using T7 and SP6 primers, with BigDye^®^ Terminator v3.1 Cycle Sequencing Kit (Applied Biosystems, UK). The GeneRacer™ Kit for full-length, RNA ligase-mediated rapid amplification of 5′ and 3′ cDNA ends (Invitrogen) was used to sequence the 5′ and 3′ untranslated region (UTR).

### Quantitative real-time RT-PCR analysis

Primers for quantitative real-time RT-PCR (RT-qPCR) were selected and optimized for all genes and variants (Additional file 2). Primers were designed overlapping exon/exon boundaries to ensure specificity for variants; these were amplified and sequenced using end-point RT-PCR to ensure specificity before performing RT-qPCR. Reactions contained 2 μL template cDNA (diluted 1/10–1/40), 7.5 μL FastStart Universal SYBR Green Master (Rox) 2 × concentration (Roche, UK), 0.2–1.0 μL of each primer at 10 mM and nuclease-free water to 15 μL. All reactions were prepared using a CAS-1200™ Precision Liquid Handling System and performed on the Rotor-Gene™ 3000 or Rotor-Gene Q (Qiagen). The amplification profile used was the same for each gene except for the annealing temperature; 5 min at 94 °C, followed by 40 cycles of 20 s at 94 °C, 20 s at optimized annealing temperature for each primer set (Additional file 2) and 20 s at 72 °C, followed by dissociation curve analysis to confirm a single gene product; amplicons were sequenced to confirm primer specificity. Relative expression levels were quantified in duplicate in three separate RT-qPCR runs, each time using cDNA from a different RT reaction, a no-template control was included in all runs. Linearity and efficiency of PCR amplification was determined for each primer pair using a standard curve generated by a dilution series of a pool of sample cDNA. All reactions had an efficiency of  >90% and correlation coefficients were R^2^ > 0.98 and a single peak melt curve.

Relative gene expression levels were calculated in GenEx 5 Standard Programme (MultiD Analyses AB, Sweden) using the comparative 2-(ΔΔ Cq) method and normalized to the geometric mean of *YWHAZ* and *SDHA.* Fold changes were calculated from ΔCq values using GenEx. The expression level in the three groups for each variant was analysed by one-way ANOVA; the difference between group means for each variant were analysed using Tukey’s multiple comparison test within one-way ANOVA, with a significance threshold of *p* ≤ 0.05.

## Results

Cloning and sequencing identified six transcript variants of *IL23R*, five variants of *IL12RB1*, a single *IL17RA* transcript and four *IL17RB* variants (Table 2).

**Table 2:** **Genbank accession numbers for the nucleotide and protein sequences of sheep IL12RB1, IL17RA, IL17RB and IL23R**

Gene	Nucleotide_id	Protein_id
*IL23R*	LN868336	CRX77112.1
*IL23Rv1*	LN868337	CRX77113.1
*IL23Rv2*	LN868338	CRX77114.1
*IL23Rv3*	LN868339	CRX77115.1
*IL23Rv4*	LN868340	CRX77116.1
*IL23Rv5*	LN868341	CRX77117.1
*IL12RB1*	LN878970	CUH82712.1
*IL12RB1v1*	LN878971	CUH82713.1
*IL12RB1v2*	LN878972	CUH82714.1
*IL12RB1v3*	LN878973	CUH82715.1
*IL12RB1v4*	LN878974	CUH82716.1
*IL17RA*	LN878979	CUH82721.1
*IL17RB*	LN878975	CUH82717.1
*IL17RBv1*	LN878976	CUH82718.1
*IL17RBv2*	LN878977	CUH82719.1
*IL17RBv3*	LN878978	CUH82720.1

### Characterization of sheep *IL23R* and *IL12RB1*

Sheep *IL23R* is encoded by 11 exons on the plus strand of chromosome 1 (NC_019458.1) and encodes a mature protein of 627 amino acids. *IL23Rv1* has a 29 bp insertion in the 5′ UTR (Chr1: 42 464 555–42 464 584) but has an identical predicted protein sequence to full length IL-23R (Additional file 3). *IL23Rv2* has a 21 bp deletion (530–551) in exon 4 (Chr1: 42 476 073–42 476 094) that results in the loss of 7 amino acids (YVVYVKS) at amino acid positions 158–164 (151–157 in IL-23Rv2), and a 2 bp insertion that causes a frame shift and a premature stop codon resulting in a protein of 287 amino acids (Additional file 3). *IL23Rv3* has a 28 bp insertion (878–906) in exon 6 (Chr1: 42 492 950–42 492 977) and is predicted to encode a truncated protein of 268 amino acids. *IL23Rv4* does not contain exon 8 (Chr1: 42 512 417–42 512 506) and encodes a 597 amino acid protein as a consequence of a 30 amino acid deletion (VPQVTMKSFQHDTQNSGLLIASIFKKHLTS); this causes the excision of the extracellular region. *IL23v5* has a deletion of exon 9 (Chr1: 42 515 314–42 515 419) and is predicted to encode a truncated protein of 356 amino acids.

Sheep *IL12RB1* is encoded by 16 exons on the plus strand of chromosome 5 (NC_019462.1) and is predicted to encode a protein of 730 amino acids. The 5′ UTR is at the 5′ end of exon 1, the extracellular region spans exons 1–13, the transmembrane region is encoded by exon 14 and the 3′ UTR is located at the 3′ end of exon 16. *IL12RB1v1* contains a deletion from the 3′ end of exon 5 to exon 8 (Chr5: 4 857 176–4 859 356), which leads to a frame shift and a truncated protein of 185 amino acids. *IL12RB1v2* contains a 651 bp deletion spanning the 3′ end of exon 1 to the 5′ end of exon 7 (Chr5: 4 853 183–4 858 549); this results in a 217 amino acid deletion at position 16–233 in the extracellular region. *IL12RB1v3* contains a 528 bp deletion from exon 3 to exon 7 (Chr5: 4 855 325–4 858 535) that causes a 176 amino acid deletion at position 51–226 within the extracellular region (Additional file 3). *IL12RB1v4* contains a deletion from the 3′ end of exon 4 to the 5′ end of exon 9 (Chr5: 4 855 845–4 860 357) causing a frame shift and a truncated protein of 163 amino acids. All four *IL12RB1* splice variants are predicted to encode proteins containing the extracellular domain only.

### Characterization of sheep *IL17RA and IL17RB*

Only one sheep *IL17RA* transcript was identified (Table 1). This is a partial sequence; the 5′ 1306 bp mapped to chromosome 3 of Oar v3.1 (NC_019460.1) with >99% identity; however the 3′ 581 bp did not map, as this region of Oar v3.1 genome assembly is incomplete. Comparison with human *IL17RA* transcript variants 1 and 2 (NM_014339.6 and NM_001289905.1) shows that the sheep gene is orthologous to human variant 1 as it contains the region encoded in humans by exon 11 (Chr3: 212 818 526–212 818 627). The RT-qPCR primers used to quantify sheep *IL17RA* transcript expression were situated in exons 4 and 5.

Sheep *IL17RB* is encoded by 11 exons on the minus strand of chromosome 19 (NC_019476.1) and is predicted to encode a mature protein of 497 amino acids. The splice variant *IL17RBv1* has a deletion of 96 bp (975–1071) that results in a 32 amino acid deletion at position 311 causing a truncated intracellular domain and the loss of the conserved TRAF6 binding motif. *IL17RBv2* has a complete deletion of exon 4 (Chr19: 47 056 667–47 054 190) that causes a frame shift and a predicted protein of only 96 amino acids. *IL17RBv3* has a deletion of exon 4 and a 177 bp insertion (651–828) at the 5′ end of exon 8 (Chr19: 47 050 145–47 049 971); this also results in a premature stop codon and a predicted protein of 96 amino acids.

### Quantification of receptor expression

RT-qPCR assays were developed and expression levels of the four cytokine receptor transcripts were quantified in the paucibacillary, multibacillary and uninfected, control animals. However only *IL23R*, *IL12RB1* and *IL12RB1v3* and *v4*, *IL17RA*, *IL17RB* and *IL17RBv2* and *v3* could be accurately quantified; expression levels of all the others were too low for accurate measurement. Positive control assays, using a template sample known to contain the individual variants were used to confirm the reliability of the assay.

**Figure 1:**
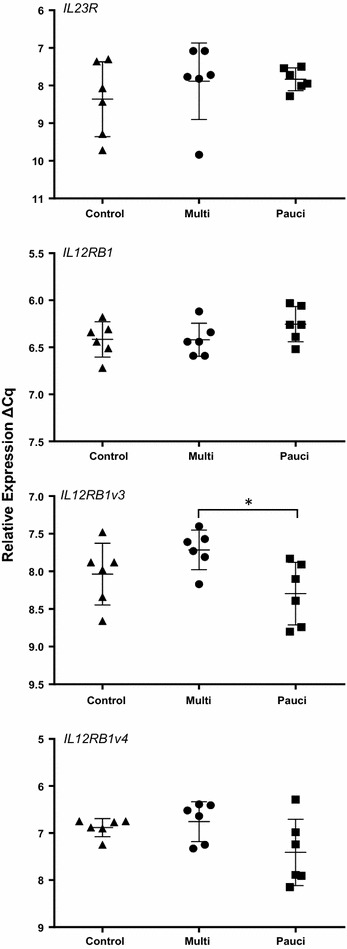
**Relative expression of** ***IL23R*** **and** ***IL12RB1*** **transcript variants.** RT-qPCR analysis of *IL23R* and *IL12RB1* transcript variants in the ICLN of paucibacillary, multibacillary and uninfected control sheep. Results are expressed as ΔCq replicate means of individual animals; error bars are ±SD of the group. *p = ≤ 0.05.

**Figure 2:**
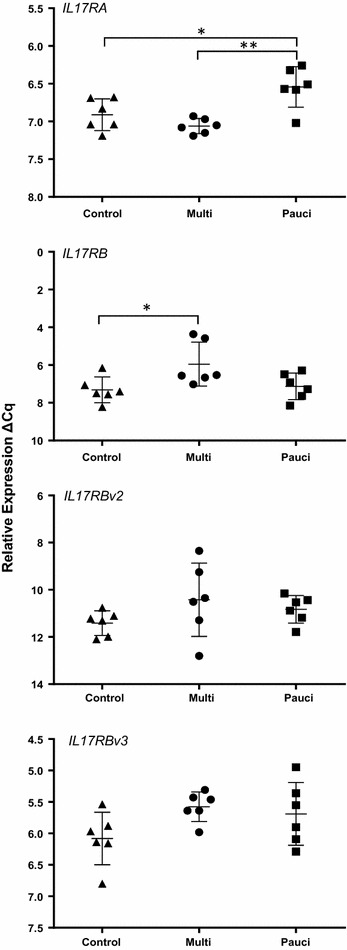
**Relative expression of** ***IL17RA*** **and** ***IL17RB*** **transcript variants.** RT-qPCR analysis of *IL17RA* and *IL17RB* transcript variants in the ICLN of paucibacillary, multibacillary and uninfected control sheep. Results are expressed as ΔCq replicate means of individual animals; error bars are ±SD of the group. *p = ≤ 0.05, **p = ≤ 0.01.

Of the *IL23R* and *IL12RB1* variants only *IL12RB1v3* showed evidence of differential expression (Figure 1) with a significant 2.1-fold (*p* = 0.04) increase in expression in the multibacillary sheep in comparison to paucibacillary animals. Of the receptors for IL-25 (Figure 2), *IL17RA* was significantly higher (Table 3) in paucibacillary sheep when compared to both multibacillary (2.0-fold, *p* = 0.001) and control sheep (2.2-fold, *p* = 0.02). Full length *IL17RB* was significantly higher in the multibacillary versus control comparison (2.49-fold, *p* = 0.04) but not in the other comparisons (although it was 2.08-fold higher in the multibacillary vs. paucibacillary comparison, but *p* = 0.08). No significant differential expression was seen with *IL17RBv2* or *v3*.

**Table 3:** **Relative expression of cytokine receptor expression within the ICLN**

Gene	ANOVA *p* value	Multi vs. control		Pauci vs. control		Multi vs. pauci	
		FC^a^	*p* value	FC	*p* value	FC	*p* value
*IL23R*	0.501	0.6	0.97	1.35	0.54	0.98	0.99
*IL12RB1*	0.232	0.97	0.99	1.57	0.28	0.62	0.28
*IL12RB1v3*	*0.049*	1.42	0.32	−1.49	0.47	*2.1*	*0.04*
*IL12RB1v4*	0.079	1.1	0.89	−1.72	0.18	1.87	0.08
*IL17RA*	*0.0016*	0.46	0.43	*2.2*	*0.02*	*−2.0*	*0.001*
*IL17RB*	*0.035*	*2.49*	*0.04*	1.19	0.93	2.08	0.08
*IL17RBv2*	0.262	1.70	0.24	1.40	0.58	1.20	0.77
*IL17RBv3*	0.104	1.60	0.12	1.52	0.24	1.09	0.88

Italics *p* ≤ 0.05.

^a^Fold change (ΔΔCq values).

## Discussion

This study is the first investigation into structural, sequence and quantitative variation of expression of genes of the IL-23 and IL-25 receptor complexes, in relation to the different pathological forms of sheep paratuberculosis. Sequencing cDNA from the ICLN of paratuberculosis-diseased sheep identified multiple transcript variants of all genes except *IL17RA*.

Splice variation is a common feature of these genes in many species including humans. Ensembl 82 [35] reports four transcript variants and three protein-coding isoforms of human *IL23R* (ENSG00000162594). However up to 24 human isoforms have been identified [36] and similar patterns can be recognised in the sheep and human variants, with four sheep variants (*IL23Rv2–v5*) predicted to encode truncated proteins. *IL23Rv2*, *v3* and *v5* lack the transmembrane and intracellular domains and are likely not to encode cell receptors but to act as antagonists as has been described for human *IL23R* variants [36]. *IL23Rv5* specifically lacks exon 10 (exon 9 in human *IL23R*) and is therefore equivalent to human *IL23RΔ9*, which is strongly linked to human IBD [26]. *IL23Rv4* encodes a protein with a truncated extracellular domain, although as with all the variants *IL23Rv4* retains the conserved WSXWS class 1 cytokine receptor activation motif [37]. However, none of the *IL23R* isoforms, including the full length form, was differential expressed in paratuberculosis. This is consistent with paratuberculosis in red deer [38], where *IL23R* showed no difference in expression in jejunal lymph nodes of animals with minimal (largely paucibacillary) or severe disease (multibacillary). Interestingly the ligand for the IL-23 receptor (IL-23A) is significantly increased in paucibacillary lesions but not in the draining lymph nodes [19]. In humans, the R318Q SNP protects against a number of immune-mediated diseases by reducing IL-23-mediated Th17 function [39]; this SNP was identified in the protein sequence translated from *IL23R*, *IL23Rv1* and *v4* nucleotide sequences but no correlation with paratuberculosis pathology was identified.

All four *IL12RB1* splice variants lack the WSXWS motif encoded within exon 7, suggesting that *IL12RB1v1*–*v4* are non-functional or that they can act as modulators of IL-12/IL-23 activity by ligand binding without signalling. A truncated *IL12RB1Δtm* variant lacking these domains has been identified in humans and mice [40]. In mice it is induced in dendritic cells (DC) after *Mycobacterium tuberculosis* infection [41]; expression promotes DC migration and may act to up-regulate IL-12RB1-dependent DC function and the immune response to mycobacteria. Mendelian susceptibility to mycobacterial diseases (immunodeficiency 30) can partly be explained by IL12RB1 deficiency; some of these patients express 5′-truncated *IL12RB1* that encodes a non-functional protein incapable of binding IL-12 or IL-23 [42]. In the paratuberculosis studies only *IL12RB1v3* was differentially expressed with a significant increase in multibacillary vs. paucibacillary sheep. This variant contains the transmembrane and intracellular domains but is 5′ truncated and does not possess the WSXWS cytokine receptor activation motif. Like the 5′ truncated human variant it may bind the cytokines but not transduce the signal, possibly contributing to the low level of IL-12/IL-23 driven Th1/Th17 activity associated with multibacillary/lepromatous pathology.

Only a single sheep *IL17RA* transcript was identified. The predicted 5′ UTR of the sheep sequence shares 62.5% identity with the 5′ end of the human coding region (BC011624.2). Consequently the predicted sheep protein is only 249 amino acids rather than 866 amino acids of the full length human IL-17RA precursor (NP_055154.3). Two splice variants have been identified in humans, the full length transcript variant 1 (NM_014339.6) of 8607 bp and encoded by 13 exons and the soluble transcript variant 2 (NM_001289905.1) of 8506 bp without exon 11, which encodes the transmembrane domain [43]. However, no functional consequences have been associated with these variations. Sheep *IL17RA* includes exon 11 and expression is significantly higher in paucibacillary disease in comparison to both multibacillary and uninfected controls. IL-17RA is a component of the receptor (with IL-17RB) for both anti-inflammatory IL-25 and (with IL-17RC) the inflammatory Th17 cytokines IL-17A and IL-17F [44]; and high levels in paucibacillary animals could confirm a role of Th17 in paratuberculosis pathology [10].

The three splice variants of sheep *IL17RB* are all predicted to encode truncated proteins. *IL17RBv2* and *v3* encode identical short extracellular proteins that do not contain a transmembrane domain or intracellular region, it is therefore unlikely that they are cellular receptors but may bind and modulate IL-25 activity. The human truncated isoform 2 (Q9NRM) [45] has an unknown function and is produced at very low levels due to a premature stop codon in the mRNA, leading to nonsense-mediated mRNA decay. *IL17RBv1* also encodes a truncated protein, with an amino-terminal truncated SEFIR (similar expression to fibroblast growth factor genes and IL-17R) domain and no TRAF6 binding motif. The SEFIR domain mediates protein interactions necessary for receptor signal transduction [46] and TRAF6 binding is essential for IL-25-mediated expression of IL-6 and TGFβ [47].

Only full length *IL17RB* was differentially expressed in the paratuberculosis diseased sheep, expression in the ICLN of the multibacillary sheep was significantly greater (2.49-fold, *p* = 0.04) than in uninfected controls. The levels in paucibacillary and control animals were not significantly different. This argues that IL-25 signalling is playing a role in multibacillary (lepromatous) pathology. IL-25 interaction with IL-17RA/IL-17RB induces the production of the pro-inflammatory cytokines IL-6 and IL-8 by endothelia [48], and IL-4 and IL-13 by T cells [49] leading to the development of Th2-associated pathology [50] especially at mucosal sites.

In conclusion, we have identified transcript variants in sheep of the four genes that make up the receptor complexes of IL-23 and IL-25; two cytokines important for the development of Th1/Th17 and Th2 T cells and in the pathogenesis of inflammatory disease at mucosal surfaces. RT-qPCR analysis of the different variants highlighted the role of the amino-terminal truncated *IL12RB1v3* and full length *IL17RB* in multibacillary pathology; and full length *IL17RA* in paucibacillary disease. This study of the gene structure, sequence and expression of IL-23 and IL-25 has provided an initial insight into the role of differential T cell activation associated with the two pathological forms of sheep paratuberculosis.

## Additional files

10.1186/s13567-016-0314-4 **Primer sequences used for amplifying full length genes.** The primer sequences and their Tm (°C) used to amplify the full length cytokine genes; and PCR product size.
10.1186/s13567-016-0314-4 **Primer sequences used for RT-qPCR.** The primer sequences and their Tm (°C) used to for relative RT-qPCR; and PCR product size.
10.1186/s13567-016-0314-4 **Derived protein sequences of cytokine transcript variants.** A. IL-23R derived protein sequences. Comparison of IL23R and four transcript variants. B. IL-12RB1 derived protein sequences. Comparison of IL12RB1 and IL12RB1v3. C. IL-17RB derived protein sequences. Comparison of IL17RB and three transcript variants.
