# Supplementary material for: Variations in IL-23 and IL-25 receptor gene structure, sequence and expression associated with the two disease forms of sheep paratuberculosis
Source: Vet Res. 2016 Feb 9;47:27. doi: 10.1186/s13567-016-0314-4 (PMC4748472; doi:10.1186/s13567-016-0314-4)
Supplement: Supplementary file 1 — 10.1186/s13567-016-0314-4 Primer sequences used for amplifying full length genes. The primer sequences and their Tm (°C) used to amplify the full length cytokine genes; and PCR product size. [file 13567_2016_314_MOESM1_ESM.pdf]

Additional File 1 Primer sequences used for amplifying full length genes

Gene	Primer	Sequence (5'-3')	Tm (°C)	Product size (bp)
IL23R	For	AAATTGACAGGCAGCAGTGAGG	55	2030
	Rev	TCTTCAGATTTCAAGGCAGGTTCT		
	For*	AAGGAAGACGTGAGGCCGATAC	62	2000
	Rev*	TTTCAAGGCAGGTTCTGACTACAC		
IL12RB1	For	ACCGGCAGCACAGAAGTTCA	55	2382
	Rev	GCCCTCTCTGAACCTCACTG		
IL17RB	For	AATAAGAGCGCGCGGCCCGAAG	55	1630
	Rev	ACTTCAGATTCTCACAGACACTT		
	For*	CGGCCCGAAGCCGATCCGGA	60	1575
	Rev*	GGGTCAGAAGGCTTTAGGG		
IL17RA	For	TTCCCGTGGTTCACATCGAG	52	539
	Rev	CAGGGTGACCGTGATGTGG		
	3' RACE	ACCGTCCACCACCTGCCTAAGCCCA	60	
	3' RACE*	ACATCACCGTGGAGACCCTTGAGG	68	
	5' RACE	TCCATGGGGTGAAGCTCAGCCGCAG	60	
	5'RACE*	CGTCAGGGATGGGCTTAGGCAGGT	68	
	5' UTR For	CTGTCTGCAAATACAAGGTCCTG	52	399
	5' UTR Rev	TCCACTCGATGTGAACCACG		
