# Supplementary material for: Variations in IL-23 and IL-25 receptor gene structure, sequence and expression associated with the two disease forms of sheep paratuberculosis
Source: Vet Res. 2016 Feb 9;47:27. doi: 10.1186/s13567-016-0314-4 (PMC4748472; doi:10.1186/s13567-016-0314-4)
Supplement: Supplementary file 2 — 10.1186/s13567-016-0314-4 Primer sequences used for RT-qPCR. The primer sequences and their Tm (°C) used to for relative RT-qPCR; and PCR product size. [file 13567_2016_314_MOESM2_ESM.pdf]

Additional File 2 Primer sequences used for RT-qPCR

Gene	Primer	Sequence (5'-3')	Tm (°C)	Product size (bp)
IL23R	For	GGTGTACGTGAAGAGTTTAGAGACAG	62	131
	Rev	CAGTACATTTGAAGCTTGGACCC		
IL23R TV1	For	ACAGGTCGTAAGAGAGCGACAAAG	58	120
	Rev	AATCCCTCCATGACACCAACTGAA		
IL23R TV2	For	CATAGACACAAATTTAGAGACAGAA	60	129
	Rev	CAGTACATTTGAAGCTTGGACCC		
IL23R TV3	For	ACTTGGAACGTAAGCTAAATTCACT	58	85
	Rev	AATTCTGACTGTTGCTCATATGTAA		
IL23R TV4	For	TCCTGAAATAGACAACGGGAAAC	58	79
	Rev	AGAACTGACAGCATAACAGCAAA		
IL23R TV5	For	ACACCTTACTTCTGAATTAAAAGAAG	58	195
	Rev	CTGGGAGAATGATTTCTATCTCTG		
IL12RB1	For	AGGATTCGGTGTCTGTGGACTGGA	61	110
	Rev	CAGACGCTTGGTTGCTGTCCTCCT		
IL12RB1 TV1	For	TATCCGTGCTCCACAATGTCACTCT	60	150
	Rev	GCAAAGCTGCTCTTGGACATCTTGA		
IL12RB1 TV2	For	CTGCTCTTCTCAAGGACCGGATGGG	60	82
	Rev	TTCAGGGGGGATGCACAAGAGGAGAA		
IL12RB1 TV3	For	CTGAGCTGGAGCAGCCCTGTGTGCAT	62	120
	Rev	GCTGCTCATGTAAGGCCACCTGCC		
IL12RB1 TV4	For	CACCATACCCAGATGCAGACTCA	61	187
	Rev	CGCAGAGCCTGTGGCAAAGA		
IL17RB	For	CGGGCAGATGTGGACGTTTTC	60	130
	Rev	TGAAGTTCACAGCCATGGAGGG		
IL17RB TV1	For	GATCATGAGAGAAGTGTGCTGGG	58	118
	Rev	GAAGTAACAAACGTGCCTCCAGA		
IL17RB TV2	For	CGGGCAGATGTGGACGTTTTC	56	129
	Rev	TGAAGTTCACAGCCATGGAGGG		
IL17RB TV3	For	TAACTCGAACTTCTGTGGTGGTCC	58	122
	Rev	GGTGCAGTGGGTCTTCAGTGAG		
IL17RA	For	CACACTGAGGCATCATCACAAGC	60	144
	Rev	GAAGTTTCTGGATTGGTGGTTTGG		
SDHA	For	ACCTGATGCTTTGTGCTCTGC	62	126
	Rev	CCTGGATGGGCTTGGAGTAA		
YWHAZ	For	TGTAGGAGCCCGTAGGTCATC	60	101
	Rev	TCTCTCTGTATTCTCGAGCCATC		
