# Supplementary material for: Variations in IL-23 and IL-25 receptor gene structure, sequence and expression associated with the two disease forms of sheep paratuberculosis
Source: Vet Res. 2016 Feb 9;47:27. doi: 10.1186/s13567-016-0314-4 (PMC4748472; doi:10.1186/s13567-016-0314-4)
Supplement: Supplementary file 3 — 10.1186/s13567-016-0314-4 Derived protein sequences of cytokine transcript variants. A. IL-23R derived protein sequences. Comparison of IL23R and four transcript variants. B. IL-12RB1 derived protein sequences. Comparison of IL12RB1 and IL12RB1v3. C. IL-17RB derived protein sequences. Comparison of IL17RB and three transcript variants. [file 13567_2016_314_MOESM3_ESM.pdf]

Additional File 3 Derived protein sequences of cytokine receptor transcript variants

A. IL-23R derived protein sequences

```
IL23R      MNQVTIHWDVVIALYIFFSWCHGGITNINCSGHIWVEPATIFRMGMNISIYCQAAIRNCQ  60
IL23Rv1    MNQVTIHWDVVIALYIFFSWCHGGITNINCSGHIWVEPATIFKMGMNISIYCQAAIRNCQ  60
IL23Rv2    MNQVTIHWDVVIALYIFFSWCHGGITNINCSGHIWVEPATIFKMGMNISIYCQAAIRNCQ  60
IL23Rv3    MNQVTIHWDVVIALYIFFSWCHGGITNINCSGHIWVEPATIFKMGMNISIYCQAAIRNCQ  60
IL23Rv4    MNQVTIHWDVVIALYIFFSWCHGGITNIDCSGHIWVEPATIFKMGMNISIYCQAAIRNCQ  60
IL23Rv5    MNQVTIHWDVVIALYIFFSWCHGGITNINCSGHIWVEPATIFKMGMNISIYCQAAIRNCQ  60
           **************************** ************* *****************

IL23R      PRKLYFYKNGIKERFHITRINKTTARLWYNNFVEPQAFVYCTAECSRYFPETLICGKDIS  120
IL23Rv1    PRKLYFYKNGIKERFHITRINKTTARLWYNNFVEPQAFVYCTAECSRYFSETLICGKDIS  120
IL23Rv2    PRKLYFYKNGIKERFHITRINKTTARLWYNNFVEPQAFVYCTAECSRYFPETLICGKDIS  120
IL23Rv3    PRKLYFYKNGIKERFHITRINKTTARLWYNNFVEPQAFVYCTAECSRYFPETLICGKDIS  120
IL23Rv4    PRKLYFYKNGIKERFHITRINKTTARLWYNNFVEPQAFVYCTAECSRYFPETLICGKDIS  120
IL23Rv5    PRKLYFYKNGIKERFHITRINKTTARLWYNNFVEPQAFVYCTAECSRYFPETLICGKDIS  120
           ************************************************* **********

IL23R      SGYPPDVPDKVTCVIYEYSGNMTCTWNPGRPTYIDTKYVVYVKSLETEEEQEYLTSSYIN  180
IL23Rv1    SGYPPDVPDKVTCVIYEYSGNMTCTWNPGRPTYIDTKYVVYVKSLETEEEQEYLTSSYIN  180
IL23Rv2    SGYPPDVPDKVTCVIYEYSGNMTCTWNPGRPTYIDTN-------LETEEEQEYLTSSYIN  173
IL23Rv3    SGYPPDVPDKVTCVIYEYSGNMTCTWNPGRPTYIDTKYVVYVKSLETEEEQEYLTSSYIN  180
IL23Rv4    SGYPPDVPDKVTCVIYEYSGNMTCTWNPGRPTYIDTKYVVYVKSLETEEEQEYLTSSYIN  180
IL23Rv5    SGYPPDVPDKVTCVIYEYSGNMTCTWNPGRPTYIDTKYVVYVKSLETEEEQEYLTSSYIN  180
           ************************************        ****************

IL23R      ISTDSLQKGKKYLVWVQASNVLGTEKSKQLQIHLDDIVIPSASIISRAEDINTTVSKTVI  240
IL23Rv1    ISTDSLQKGKKYLVWVQASNVLGTEKSKQLQIHLDDIVIPSASIISRAEDINTTVSKTVI  240
IL23Rv2    ISTDSLQKGKKYLVWVQASNVLGTEKSKQLQIHLDDIVIPSASIISRAEDINTTVSKTVI  233
IL23Rv3    ISTDSLQKGKKYLVWVQASNVLGTEKSKQLQIHLDDIVIPSASIISRAEDINTTVSKTVI  240
IL23Rv4    ISTDSLQKGKKYLVWVQASNVLGTEKSKQLQIHLDDIVIPSASIISRAEDINTTVSKTVI  240
IL23Rv5    ISTDSLQKGKKYLVWVQASNVLGTEKSKQLQIHLDDIVIPSASIISRAEDINTTVSKTVI  240
           ************************************************************

IL23R      HWNSQTSIEKVSCEMRYKATTNQTWNVKEFDTNFTYEQQSEFYLQPNATYVFQVRCQETG  300
IL23Rv1    HWNSQTSIEKVSCEMRYKATTNQTWNVKEFDTNFTYEQQSEFYLQPNATYVFQVRCQETG  300
IL23Rv2    HWNSQTSIEKVSCEMRYKATTNQTWNTLKNLIPILHMSNSQNSTCSQ--------MLHTY  285
IL23Rv3    HWNSQTSIEKVSCEMRYKATTNQTWNVS--------------------------------  268
IL23Rv4    HWNSQTSIEKVSCEMRYKATTNQTWNVKEFDTNFTYEQQSEFYLQPNATYVFQVRCQETG  300
IL23Rv5    HWNSQTSIEKVSCEMRYKATTNQTWNVKEFDTNFTYEQQSEFYLQPNATYVFQVRCQETG  300
           **************************

IL23R      KRYWQPWSSPFFHKTPEIVPQVTMKSFQHDTQNSGLLIASIFKKHLTSDNGKQDIGLLLG  360
IL23Rv1    KRYWQPWSSPFFHKTPEIVPQVTVKSFQHDTQNSGLLIASIFKKHLTSDNGKQDIGLLLG  360
IL23Rv2    FK----------------------------------------------------------  287
IL23Rv3    ------------------------------------------------------------  268
IL23Rv4    KRYWQPWSSPFFHKTPEI------------------------------DNGKQDIGLLLG  330
IL23Rv5    KRYWQPWSSPFFHKTPEIVPQVTMKSFQHDTQNSGLLIASIFKKHLTSELKEESYC----  356

IL23R      MVFFAVMLSVLSLFGIFNKSLRTGIKRRILLLIPKWLYEDIPKLENSKVVKILQEKNEFM  420
IL23Rv1    MVFFAVMLSVLSLFGIFNKSLRTGIKRRILLLIPKWLYEDIPKLENSKVVKILQEKNEFM  420
IL23Rv2    ------------------------------------------------------------  287
IL23Rv3    ------------------------------------------------------------  268
IL23Rv4    MVFFAVMLSVLSLFGIFNKSLRTGIKRRILLLIPKWLYEDIPKLENSKVVKILQEKNEFM  390
IL23Rv5    ------------------------------------------------------------  354

IL23R      NNNSSEQVLYVDPVITEIEIILPEEKPIGYKKENNTGCLERKESLEKSLLTNTTVVYIPD  480
IL23Rv1    NNNSSEQVLYVDPVITEIEIILPEEKPIGYKKENNTGCLERKESLEKSLLTNTTVVYIPD  480
IL23Rv2    ------------------------------------------------------------  287
IL23Rv3    ------------------------------------------------------------  268
IL23Rv4    NNNSSEQVLYVDPVITEIEIILPEEKPIGYKKENNTGCLERKESLEKSLLTNTTVVYIPD  450
IL23Rv5    ------------------------------------------------------------  354
```

```
IL23R        LNTGYKPQISSFLPGGNHLRNDDETASSILEPPADSLNLGNNARFKKYPDFAFSVSSTNS  540
IL23Rv1      LNTGYKPQISSFLPGGNHLRNDDETASSILEPPADSLNLGNNARFKKYPDFAFSVSSTNS  540
IL23Rv2      ------------------------------------------------------------  287
IL23Rv3      ------------------------------------------------------------  268
IL23Rv4      LNTGYKPQISSFLPGGNHLRNDDETASSILEPPADSLNLGNNARFKKYPDFAFSVSSTNS  510
IL23Rv5      ------------------------------------------------------------  354


IL23R        LSNTLFLEELSLILNQGECSPPDMQNSIEGETAMLLEDALLNETIPEQTLLPDEFVSCLG  600
IL23Rv1      LSNTLFLEELSLILNQGECSPPDMQNSIEGETAMLLEDALLNETIPEQTLLPDEFVSCLG  600
IL23Rv2      ------------------------------------------------------------  287
IL23Rv3      ------------------------------------------------------------  268
IL23Rv4      LSNTLFLEELSLILNQGECSPPDMQNSIEGETAMLLEDALLNETIPEQTLLPDEFVSCLG  570
IL23Rv5      ------------------------------------------------------------  356


IL23R        SMNKELPSINSYFPQNILESHFNRFHS 627
IL23Rv1      SMNKELPSINSYFPQNILESHFNRFHS 627
IL23Rv2      --------------------------- 287
IL23Rv3      --------------------------- 268
IL23Rv4      SMNKELPSINSYFPQNILESHFNRFHS 597
IL23Rv5      --------------------------- 356
```

Bold, class 1 cytokine receptor activation motif
Box, R381Q variant site
Underline, transmembrane domain

A. IL-12RB1 derived protein sequences

```
IL12RB1          1 MGQWGFRLVAFLLLLCHRQGAEACGTVGCCFQNPPYPDADSGSASGPRAL     50
                   ||||||||||||||||||||||||||||||||||||||||||||||||||
IL12RB1v3        1 MGQWGFRLVAFLLLLCHRQGAEACGTVGCCFQNPPYPDADSGSASGPRAL     50

IL12RB1         51 SCYRLSSSAGYECSWEYEGPAAGVIHFLRCCLQSGRCCFFATGSATKLQF    100

IL12RB1v3       51 --------------------------------------------------     50

IL12RB1        101 SDQDGISVLHNVTLWVESRAANRTEKSPNVTLNLYSSVKYDPPPGNIKVS    150

IL12RB1v3       51 --------------------------------------------------     50

IL12RB1        151 KSAGKLRMEWETPARQDGAEVQFRHRTPGSPWKLGDCGRQDDAGFESCLC    200

IL12RB1v3       51 --------------------------------------------------     50

IL12RB1        201 PLEMDMAQEFQLRRRLGPGVPGGPWSSWSSPVCIPPETPPQAEVRFSAEQ    250
                                             ||||||||||||||||||||||||
IL12RB1v3       51 --------------------------SWSSPVCIPPETPPQAEVRFSAEQ     74

IL12RB1        251 LCPDGRRQVALHEQLPQLELPEGCLGPDPGMEVTYKIHLHMLSCPCKTRA    300
                   ||||||||||||||||||||||||||||||||||||||||||||||||||
IL12RB1v3       75 LCPDGRRQVALHEQLPQLELPEGCLGPDPGMEVTYKIHLHMLSCPCKTRA    124

IL12RB1        301 KKTLRLKRKLVLSSAAYDLTIVSQNRFGLGPNQTWRIPACIHSEPGLLNI    350
                   ||||||||||||||||||||||||||||||||||||||||||||||||||
IL12RB1v3      125 KKTLRLKRKLVLSSAAYDLTIVSQNRFGLGPNQTWRIPACIHSEPGLLNI    174

IL12RB1        351 SAGANGTTMHWPARAQGMRYCIEWQLQGQEENLAACIVTAPQDADAAGMA    400
                   ||||||||||||||||||||||||||||||||||||||||||||||||||
IL12RB1v3      175 SAGANGTTMHWPARAQGMRYCIEWQLQGQEENLAACIVTAPQDADAAGMA    224

IL12RB1        401 THSWSQESGALAQKACYHIAIFASAHPEKLTSWSTVLSTYHFGGNASAAG    450
                   ||||||||||||||||||||||||||||||||||||||||||||||||||
```

```
IL12RB1v3        225 THSWSQESGALAQKACYHIAIFASAHPEKLTSWSTVLSTYHFGGNASAAG    274

IL12RB1          451 SPQHVSVRKLSQDSVSVDWTPSLLSTCPGVLKEYVVYFQEEDSNQASELH    500
                     ||||||||||||||||||||||||||||||||||||||||||||||||||
IL12RB1v3        275 SPQHVSVRKLSQDSVSVDWTPSLLSTCPGVLKEYVVYFQEEDSNQASELH    324

IL12RB1          501 VKPTETQVTLQGLRAGTAYKVQVRADTAKWRGAWSQPLHFTIEVQVSELS    550
                     ||||||||||||||||||||||||||||||||||||||||||||||||||
IL12RB1v3        325 VKPTETQVTLQGLRAGTAYKVQVRADTAKWRGAWSQPLHFTIEVQVSELS    374

IL12RB1          551 DLSIFLASLGSFVSILLLGIFGYLSLNRAVRHLCPPLPTPGASTAIKFSG    600
                     ||||||||||||||||||||||||||||||||||||||||||||||||||
IL12RB1v3        375 DLSIFLASLGSFVSILLLGIFGYLSLNRAVRHLCPPLPTPGASTAIKFSG    424

IL12RB1          601 SQGKQVWQWTSPADFPEEVSPHEALMVNISWEKGEGADMGTLGTLKEKME    650
                     ||||||||||||||||||||||||||||||||||||||||||||||||||
IL12RB1v3        425 SQGKQVWQWTSPADFPEEVSPHEALMVNISWEKGEGADMGTLGTLKEKME    474

IL12RB1          651 LPLRAPKPAPDTELPLKDRKQMQGCPEAGALRPGWQDGLVEDSLAQVARP    700
                     ||||||||||||||||||||||||||||||||||||||||||||||||||
IL12RB1v3        475 LPLRAPKPAPDTELPLKDRKQMQGCPEAGALRPGWQDGLVEDSLAQVARP    524

IL12RB1          701 PLLLLGGLRQAPRFGSQGEAGTAASSYRED    730
                     ||||||||||||||||||||||||||||||
IL12RB1v3        525 PLLLLGGLRQAPRFGSQGEAGTAASSYRED    554
```

Bold, class 1 cytokine receptor activation motif
Underline, transmembrane domain

B. IL-17RB derived protein sequences

```
IL17RB        MLLVLLSLAALCWGAVPPEPTIQCGSEPGPSPEWMVRHTLTPGDLRDLRVEPIKSSVDLE 60
IL17RBv1      MLLVLLSLAALCWGAVPPEPTIQCGSEPGPSPEWMVRHTLTPGDLRDLRVEPIKSSVDLE 60
IL17RBv2      MLLVLLSLAALCWGAVPPEPTIQCGSEPGPSPEWMVRHTLTPGDLRDLRVEPIKSSVDLE 60
IL17RBv3      MLLVLLSLAALCWGAVPPEPTIQCGSEPGPSPEWMVRHTLTPGDLRDLRVEPIKSSVDLE 60
              ************************************************************

IL17RB        DSSILMNISWILRADASIRLLKATKICVMGKSHFQSRSCIRCNYTQAFRTQTRHSGGKWT 120
IL17RBv1      DSSILMNISWILRADASIRLLKATKICVMGKSHFQSRSCIRCNYTQAFRTQTRHSGGKWT 120
IL17RBv2      DSSILMNISWILRADVDVFLHWF--SCRAEHSLFHWSP---------------------- 96
IL17RBv3      DSSILMNISWILRADVDVFLHWF--SCRAEHSLFHWSP---------------------- 96
              ***************..: *       *    :* *:

IL17RB        FSYIGFPVELNTVYFIGAHNIPNANMNEDGPSMAVNFTSPGCLDHVMKYKKKCIEAGSLW 180
IL17RBv1      FSYIGFPVELNTVYFIGAHNIPNANMNEDGPSMAVNFTSPGCLDHVMKYKKKCIEAGSLW 180
IL17RBv2      ------------------------------------------------------------ 96
IL17RBv3      ------------------------------------------------------------ 96

IL17RB        KPNITACKKSANTVEVNFTTSPLGDRYMALIQNTSVIGTSYVSEKELTRTSVVVHVTGES 240
IL17RBv1      KPNITACKKSANTVEVNFTTSPLGDRYMALIQNTSVIGTSYVSEKELTRTSVVVHVTGES 240
IL17RBv2      ------------------------------------------------------------ 96
IL17RBv3      ------------------------------------------------------------ 96

IL17RB        EGAVVQLTPYFHTCGNDCIRQRGTVVRCPQTGVSPQDHERSVLGGWLPLLLSALLVATWV 300
IL17RBv1      EGAVAQLTPYFHTCGNDCIRQRGTVVRCPQTGVSPQDHERSVLGGWLPLLLSALLVATWV 300
IL17RBv2      ------------------------------------------------------------ 96
IL17RBv3      ------------------------------------------------------------ 96

IL17RB        LVAGIYLIWRHERIKKTSFSTTTLLPSLKVLVVYPSEICFHHTVCYFTEFLQNRCRSEVI 360
IL17RBv1      LVAGIYLIWRH--------------------------------VCYFTEFLQNRCRSEVI 328
IL17RBv2      ------------------------------------------------------------ 96
```

```
IL17RBv3        ---------------------------------------------------------------- 96

IL17RB          LEKWQKKKIAEMGPVQWLTTQKQAADKVIFLLSNDNTMCDGTCDKKEGGPCENSQDLFHL 420
IL17RBv1        LEKWQKKKIAEMGPVQWLTTQKQAADKVIFLLSNDNTMCDGTCDKKEGGPCENSQDLFHL 388
IL17RBv2        ---------------------------------------------------------------- 96
IL17RBv3        ---------------------------------------------------------------- 96

IL17RB          AFNLFCSDLRSQTHLRKYVVVYFREGDIKDSYSALSVCPTYRLTKDATDFCAELLHAKQH 480
IL17RBv1        AFNLFCSDLRSQTHLRKYVVVYFREGDIKDSYSALSVCPTYRLTKDATDFCAELLHAKQH 448
IL17RBv2        ---------------------------------------------------------------- 96
IL17RBv3        ---------------------------------------------------------------- 96

IL17RB          VSVGRRSRARHYSCLSL 497
IL17RBv1        VSVGRRSRARHYSCLSL 465
IL17RBv2        ----------------- 96
IL17RBv3        ----------------- 96
```

Bold, SEFIR domain
Box, TRAF6 binding site
Underline, transmembrane domain
